# Analysis of intra-genomic GC content homogeneity within prokaryotes

**DOI:** 10.1186/1471-2164-11-464

**Published:** 2010-08-06

**Authors:** Jon Bohlin, Lars Snipen, Simon P Hardy, Anja B Kristoffersen, Karin Lagesen, Torunn Dønsvik, Eystein Skjerve, David W Ussery

**Affiliations:** 1 Norwegian School of Veterinary Science, Department of Food Safety and Infection Biology, Ullevålsveien 72, P.O. Box 8146 Dep, NO-0033 Oslo, Norway; 2 National Veterinary Institute, Section of epidemiology, Ullevålsveien 68, Pb 750 Sentrum, N-0106 Oslo, Norway; 3 Norwegian University of Life Sciences, Department of Chemistry, Biotechnology and Food Sciences, Ås, Norway; 4 University of Oslo, Department of Informatics, Pb. 1080, 0316 Oslo, Norway; 5 Center for Biological Sequence Analysis, Department of Systems Biology, Comparative Genomics Unit, Technical University of Denmark, DK-2800 Lyngby, Denmark; 6 Centre for Molecular Biology and Neuroscience (CMBN), Institute of Medical Microbiology, Rikshospitalet, NO-0027 Oslo, Norway

## Abstract

**Background:**

Bacterial genomes possess varying GC content (total guanines (Gs) and cytosines (Cs) per total of the four bases within the genome) but within a given genome, GC content can vary locally along the chromosome, with some regions significantly more or less GC rich than on average. We have examined how the GC content varies within microbial genomes to assess whether this property can be associated with certain biological functions related to the organism's environment and phylogeny. We utilize a new quantity *GCVAR*, the intra-genomic GC content variability with respect to the average GC content of the total genome. A low *GCVAR *indicates intra-genomic GC homogeneity and high *GCVAR *heterogeneity.

**Results:**

The regression analyses indicated that *GCVAR *was significantly associated with domain (i.e. archaea or bacteria), phylum, and oxygen requirement. *GCVAR *was significantly higher among anaerobes than both aerobic and facultative microbes. Although an association has previously been found between mean genomic GC content and oxygen requirement, our analysis suggests that no such association exits when phylogenetic bias is accounted for. A significant association between *GCVAR *and mean GC content was also found but appears to be non-linear and varies greatly among phyla.

**Conclusions:**

Our findings show that *GCVAR *is linked with oxygen requirement, while mean genomic GC content is not. We therefore suggest that *GCVAR *should be used as a complement to mean GC content.

## Background

The knowledge of the chemical basis for nucleic acids goes back more than a hundred years, to the work of Miescher [1]. By the early 1950's, it was known that the relative frequency of the four DNA bases ("base composition") was different for different organisms [2], and in general the number of A's was equal to the number of T's, and the number of G's was the same as the number of C's; this is known as 'Chargaff's first parity rule' [3]. Further, for nearly all genomes studied, the parity rule appears to extend to each strand of the chromosome, when averaged over long distances [4], although in bacterial chromosomes, there is a clear bias of G's towards the replication leading strand, and for some genomes (many Firmicutes, for example) the A's are also biased towards the leading strand [5]. For a circular chromosome with the replication origin and terminus on exactly opposite sides, this bias of G's towards the replication leading strand will average out to near zero, when one only looks at the DNA sequence in the GenBank file, and the sequence will appear to conform to Chargaff's second rule.

From the Genbank database at NCBI http://www.ncbi.nlm.nih.gov/genomes/lproks.cgi it can be seen that GC content in prokaryotes ranges from 16.6% in *Carsonella ruddii *strain Pv to 74.9% in *Anaeromyxobacter dehalogenans *Strain 2CP-C. Within a given genome, the GC content along the chromosome can vary, although since most bacterial genomes have a high coding density, usually the variation is less than that found in eukaryotes [6]. The average genomic GC content is an important property in microbial genomes and has been associated with properties such as genome size [7], oxygen, and nitrogen exposure [8,9] and specific habitats [10-13]. For instance, intracellular bacteria have, on average, smaller genomes and are mostly AT rich, while soil bacteria tend to have larger genomes and higher %GC [14]. Higher AT content in intracellular bacteria may be attributed to a loss of repair genes; this loss will eventually lead to an increase in mutation rates from cytosine to thymine [15,16]. Genes not expressed will eventually lead to reduced genome sizes [15,16]. Higher GC content in soil bacteria may be due to the increased availability of nitrogen [9]. However, increased nitrogen in the soil does not explain why GC rich bacteria often have larger genomes. The base composition in GC rich genomes might reflect stronger selective forces than AT rich genomes [17-19]. This may indicate that GC rich microbes live in more complex environments than intracellular bacteria [20]. The reasons for stronger selective forces and GC richness is not known, but may be connected to the fact that considerably more energy is required to de-stack GC rich DNA sequences than AT rich DNA sequences [21].

Although GC content has been found to vary only slightly within prokaryotic genomes some regions differ more than others. A large region flanking the replication origin, for instance, is more GC rich than the average genomic GC content [22] whereas the region around replication terminus is more AT rich [5]. Surface proteins and RNA genes often have GC content that differs from the average genomic GC content [22], and protein coding regions have been found to be, on average, approximately 5% more GC rich than non-coding regions [18]. In addition to being more GC rich, coding regions have been found to be more homogeneous in terms of base composition than non-coding regions [18]. The GC heterogeneity in coding regions has, however, been found to be associated with mean genomic AT content in non-coding regions [18,23]. In other words, GC content variability tends to increase with higher mean genomic AT content in non-coding regions.

Horizontally transferred DNA may have a different fraction of GC than the host genome as a result of different evolutionary pressures [6,24-26]. Since horizontally transferred DNA is often linked to pathogenesis in microbes [27], detection of such regions is of great importance. The GC content of foreign DNA will, however, become progressively more similar to the host genome in a process known as amelioration [24] making such regions more difficult to detect as time progress [25]. The conformation of base compositional patterns from foreign DNA to host DNA may be related to the finding that a particular subunit of the DNA polymerase III, the Pol III α subunit, appears to be driving genomic GC content in prokaryotes [28].

There is a considerable amount of research and documentation related to mean genomic GC content in prokaryotes demonstrating that this property is the result of many factors interacting in a highly complex manner [29]. On the other hand, analysis of genomic GC content variability within microbial chromosomes, has received much less attention. A more recent overview of methodology used to analyze GC content variation within genomes can be found in Bernaola-Galvan *et. al*., [30], and a study of how intra-genomic GC content variation affects codon usage is described by Daubin *et. al*. [31]. In the present work, we introduce the *GCVAR *measure to examine GC content variability within prokaryotic genomes. The *GCVAR *metric gives a measure of how GC content varies within a given genome with respect to the mean genomic GC content. A low *GCVAR *thus points to little GC content variation, or GC content homogeneity, within the genome, while a high *GCVAR *designates varying GC content, or GC content heterogeneity.

To the best of our knowledge, no study has examined the interplay between environmental factors and GC content homogeneity in prokaryotes. In the present study the aim was therefore to examine whether GC content homogeneity in prokaryotes, measured here using the *GCVAR *measure, could be related to specific factors in the environment such as temperature and oxygen, as well as the broader properties implicated in phylogeny and GC content. To do this, regression analyses were performed using *GCVAR *as the response variable. The response variable was fitted to the following variables: oxygen requirement (a categorical variable defined as either aerobic, anaerobic or facultative), phylum, genomic GC content, genome size, growth temperature (a categorical variable used to define psychrophiles, mesophiles and thermophiles), pathogenicity (a dichotomous variable describing whether the microbe is pathogenic or not) and habitat (a categorical variable describing the environment where the microbe is found, i.e. aquatic, host-associated, multiple, specialized and terrestrial). The dataset consisted of 488 genomes (526 chromosomes) with similar strains and species removed from the analysis to reduce phylogenetic bias.

## Results and Discussion

### GC distribution within genomes

**Figure 1:**
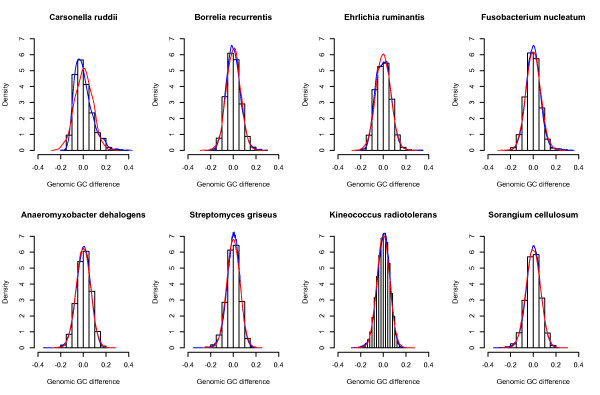
**The distributions of GC difference within genomes**. The histograms show the distribution of GC difference, *D*_*i*_, (Equation (1) in the Methods section) for eight different microbial genomes. The blue curves are empirical density estimates, while the red curves are Gaussian densities using based on the same means and standard deviations as the empirical estimates. The upper panels show the statistical distributions for four AT rich genomes, while the lower panels show the distributions for four GC rich genomes.

The histograms in Figure 1 shows the statistical distributions of GC content differences, *D*_*i *_= *GC*_*i *_- *GC *(Equation (1), Methods section), within four AT-rich and four GC-rich genomes. The statistical distributions shown in Figure 1 are based on the differences, or residuals, between the GC content of a 100 bp non-overlapping sliding window and mean genomic GC content for each of the 8 genomes. Figure 1 therefore shows the statistical distributions of how GC contents differences are distributed within each of the described genomes. With the exception of *Carsonella rudii*, one of the smallest bacterial genomes currently sequenced (~160 kbp), all empirical distributions follow the bell shaped Gaussian curve. This indicates that GC difference within prokaryotic genomes appears to be a sum of many independent processes, giving a Gaussian like distribution according to the central limit theorem (see, for instance, [32]). Thus, it seems likely that for most prokaryotic genomes intra-genomic GC content variation appears to follow a random, white-noise like pattern, devoid of any complex and long-range interacting factors.

### The *GCVAR *regression model

**Figure 2:**
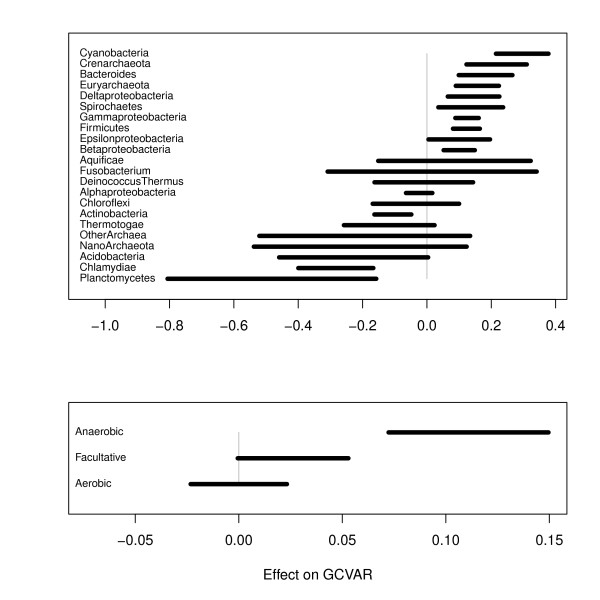
**Significant effects on GC variation**. The bars indicate 95% confidence intervals for the effects of various phyla (top panel) and oxygen requirements (lower panel) based on the regression model described by Equation (3) in the Methods section. Note that the values on the horizontal axis are scaled differently in the two panels. Categories with non-overlapping intervals can be said to differ significantly at a 5% level. Only significant effects are included.

We define *GCVAR *as a measure of the intra-genomic GC variation in a genome. A linear regression model was fitted to data for 526 prokaryote chromosomes with *GCVAR *as the response and with GC content, size, phylum, oxygen requirement, growth temperature, pathogenicity and habitat as covariates (Equation (3) in the Methods section). The results of the *GCVAR *regression model can be observed in Table 1, and in Figure 2 we show the 95% confidence intervals for the significant effects. The variables: size, growth temperature, pathogenicity and habitat had no significant influence on *GCVAR*, and were therefore discarded from further analyses.

**Table 1:** The coefficient estimates from the *GCVAR *regression model

**Factor**	**Category**	**Chromosomes**	**Average %GC**	**Average size (mbp)**	**Coefficient estimate**	***p*-value**
Phylum	Acidobacteria	2	60	7.8	-0.23	0.05
Phylum	Actinobacteria	42	66	4.8	-0.11	0.003
Phylum	Bacteroides	16	44	3.6	0.18	<0.001
Phylum	Betaproteobacteria	64	64	3.4	0.1	0.002
Phylum	Chlamydiae	8	43	1.9	-0.28	<0.001
Phylum	Crenarchaeota	16	48	2	0.22	<0.001
Phylum	Cyanobacteria	17	48	4.4	0.3	<0.001
Phylum	Deltaproteobacteria	18	58	4.7	0.15	0.001
Phylum	Epsilonproteobacteria	12	38	1.9	0.1	0.04
Phylum	Euryarcheota	31	46	2.4	0.16	<0.001
Phylum	Firmicutes	89	37	2.6	0.12	<0.001
Phylum	Gammaproteobacteria	92	47	3.7	0.12	<0.001
Phylum	Planctomycetes	1	55	7.2	-0.48	0.002
Phylum	Spirochaetes	11	37	1.7	0.14	0.01
Oxygen	Anaerobic	-	-	-	0.11	<0.001
GC	-	-	-	-	0.37	<0.001

The variable *GC *is continuous while phylum and oxygen are categorical variables. Note that for the phylum variable we have used the sum-to-zero parameterization, i.e. all estimated effects are deviations from the mean phylum effect. For the oxygen requirement variable however, we used a relative parameterization where the category "aerobic" is the reference, i.e. the estimated effect is the deviation from the aerobic effect. In addition, the number of chromosomes, average %GC, and average genomes size in mbp, are included for each phylogenetic group.

### *GCVAR *in phyla

Table 1 shows that *GCVAR *is significantly influenced by phylum. We find that 10 phyla have *GCVAR *significantly above the average phylum, and 4 phyla have *GCVAR *significantly below average. The two phyla, *Crenarchaeota *and *Euryarcheota*, (both archaea) are among the groups with an above average *GCVAR*. The archaea domain, consisting predominantly of organisms living in extreme environments, had a significantly higher *GCVAR *than bacteria (*p < 0.001*). The highest *GCVAR *are found in the aquatic group *Cyanobacteria*, which is largely populated with species capable of photosynthesis [33]. The lowest *GCVAR *are found in the phylum of the aquatic *Planctomycetes*, but this group is only based on one single genome, therefore no conclusions can be assumed at the phylum level.

### Environmental factors and phylogenetic bias

To examine how *GCVAR *was affected by phylogenetic bias a regression model similar to the one described above was fitted, i.e. *GCVAR *was the response variable, with mean genomic GC content, oxygen requirement, habitat, optimal growth temperature, and genome size as predictors. In addition, an interaction term between GC content and phylum was added to account for more similar GC content within phyla (Equation (5) in the Methods section). Using this regression model we found that oxygen requirement was the only significant factor (*p < 0.001*). *GCVAR *was significantly higher in the genomes of anaerobic microbes (103 chromosomes) as compared to the genomes of aerobic microbes, meaning that the genomes of anaerobic microbes tend to have a more heterogeneous distribution of GC content than genomes of aerobic microbes (246 chromosomes). Facultative microbes were found to have *GCVAR *values in the region between aerobic and anaerobic microbes (see Figure 2).

### The associations between mean genomic GC content, *GCVAR *and oxygen requirement

The regression models described above indicates that aerobic microbes have genomes with more homogeneous GC content than those of organisms with facultative and anaerobic oxygen requirement (see Figure 2). It has been shown that GC rich genomes tend to be more homogeneous in terms of base composition than AT rich genomes [18,19,34]. Aerobic microbes have been associated with GC rich genomes [8]. This result is supported by our linear regression model only when we ignore phylogenetic bias is (*p < 0.001*). However, adding phyla as a predictor (Equation (5) in the Methods section) fails to demonstrate such an association (*p ~0.9*).

**Figure 3:**
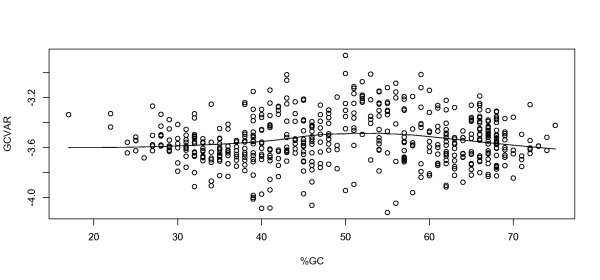
**Overall relation between GC content and *GCVAR***. The Figure shows *GCVAR *on the vertical axis versus %GC on the horizontal axis. The trend line is made using standard loess smoother.

We found that mean genomic GC content was associated with *GCVAR*, but there was no linear relationship between mean genomic GC content and *GCVAR *(Figure 3), although this does not exclude a non-linear relationship.

### *GCVAR *and DNA uptake

There are many indications that mean genomic GC content is as much affected by the environment as by phyla [10-13]. It is also well known that chromosomally integrated foreign DNA may differ in base composition as compared to host DNA. The difference in base composition between foreign and host DNA is assumed to be the result of exposure to different selective pressures. It is thought that such genetic regions may be acquired from horizontal transfer or other means of DNA uptake [6,24-26]. Since pathogenesis is often associated with horizontally transferred DNA, i.e. pathogenicity islands, [27], establishing a link between any genomic property and horizontal transfer is of considerably interest. However, no significant association (*p ~ 0.25*) was found between the dichotomous pathogenicity factor and *GCVAR *using the regression model that included all covariates discussed above (Equation (3) in the Methods section).

### Base composition and oxygen requirement

The introduction of atmospheric oxygen is presumed to have had profound effects on environment and life [35]. Increase in atmospheric oxygen is believed to have influenced cellular compartmentalization and thus to have been instrumental in the evolution of eukaryotes [36]. Prokaryotes were also affected by the introduction of oxygen [35] in that while some remained anaerobic others adapted to an aerobic metabolism [37].

The precise effect of increase in atmospheric oxygen on prokaryotic genomes is debated [37,38]. A negative correlation has been found between proteomic oxygen content and genomic GC content [37]. Although it has been suggested that genomic GC content is also affected by an aerobic lifestyle [8], the effects on prokaryote genome composition has remained unclear [37,38]. Indeed, our own results presented above do not support any connection between genomic GC content and aerobiosis. Our results did, however, find a significant association between *GCVAR *and oxygen requirement. This greater GC content homogeneity found in aerobes implies that the genomes of these organisms have been subjected to stronger selective pressures than the genomes of anaerobes. This is supported by the recent report that metabolic networks of aerobic bacteria are more complex than those of anaerobic bacteria [35]. From Figure 2 it can be seen that *GCVAR *appears to be progressively decreasing in facultative and aerobic prokaryotes, respectively.

## Conclusion

In summary, we found that *GCVAR *was associated with oxygen requirement. It is possible that *GCVAR *is associated with GC content, but from Figure 3 it appears to be a highly non-linear relationship. Other factors such as genome size, habitat and growth temperature were not found significant in the *GCVAR *model. *GCVAR *was however found to be higher in archaea than bacteria. By adding an interaction term to model the closer similarity between the genomes in the same phylogenetic group, we found that oxygen requirement was not significantly associated with mean genomic GC content in microbes.

The different results obtained for the models describing *GCVAR *and mean genomic GC content imply that these properties are governed by different influences, or are interrelated in a non-linear manner. Thus, our findings suggest that GCVAR is linked with oxygen requirement, while mean genomic GC content is not.

## Methods

All genomes and related information were gathered from the NCBI web site http://www.ncbi.nlm.nih.gov/genomes/lproks.cgi. The statistical package R[39] was used for statistical analyses and graphical representations.

### The *GCVAR *measure

To calculate GC variation within a prokaryotic genome, the number of guanine and cytosine nucleotides in a chromosome were counted and divided by chromosome size, giving the mean chromosomal GC content *GC*. A similar counting was performed for all 100 bp non-overlapping windows along the chromosome, giving the mean GC content *GC*_*i *_for window *i*. The difference, *D*_*i*_, between the mean GC content of window *i*, and the mean chromosomal GC content, can therefore be written as:(1)

The quantity *GCVAR *is then defined as the log-transformed average of the absolute value of the difference between the mean GC content of each non-overlapping sliding window *i *and mean chromosomal GC content:(2)

*N *is the maximum number of non-overlapping 100 bp sliding windows that can fit into the chromosome that is being analyzed. The log-transformation makes *GCVAR*s empirical distribution more Gaussian-like, for convenience in subsequent linear regression model fitting and statistical inference. Since the optimal sized sliding window varies from genome to genome [18], different window lengths were tested. The sliding window width of 100 bp was chosen to make the test as sensitive as possible. The other sliding window lengths tested contained 500, 1000, and 2000 bp. The 100 bp sliding window was found to be large enough to carry genome specific information without discarding weak genomic signals as noise. Since the aim of this study was to examine GC content difference within genomes, non-overlapping sliding windows were used to avoid bias and interactions from neighboring genetic regions.

### Linear models

Linear regression analysis was used to examine influences affecting *GCVAR*. In our first analysis we made a regression of *GCVAR *onto *GC *and *Size *(genome size in Mb), also including the categorical variables phylum (22 phylogenetic groups), required oxygen (aerobic, facultative, anaerobic), growth temperature (psychrophilic, mesophilic and thermophilic), pathogenicity (pathogenic, non-pathogenic) and habitat (aquatic, host-associated, multiple, specialized and terrestrial) as predictors or explanatory variables. The model can be written as:(3)

where *μ *is the overall intercept, *α*_*y*_*, y = 1,...,22*, are the effects of phylum*, δ*_*o*_*, o = 1,2,3*, are the effects of oxygen requirement*, κ*_*t*_*, t = 1,2,3*, are the effects of growth temperature*, λ*_*a*_*, a = 1,2*, are the effects of pathogenicity and *η*_*h*, _*h = 1,...4*, are the effects of habitat. *β *and *γ *are the regression coefficients for the continuous variables *GC *and *Size*, respectively.

Based on the inference using the regression model described by (3) we eliminated the non-significant variables and obtained a reduced set of predictors: *GC*, phylum and oxygen requirement. In this reduced model, we included phyla only as an interaction with *GC*. The reason for this is that genomes within the same phylum tend to have similar GC content. Hence, a main effect of phylum may actually be a phylum-dependent GC effect. The model formulated as follows:(4)

The *α*_*y *_in this model are defined as regression coefficients for each of the 22 phylum categories.

To test for possible associations between aerobiosis and mean genomic GC content, a regression model was fitted with GC as the response variable and aerobiosis as a group variable:(5)

*μ, α*_*y*_*, δ*_*o *_are the same effects as those described for Equation (3).

## Authors' contributions

JB wrote the paper and carried out analyses. LS, ABK, JB, ES carried out statistical analyses. TD suggested the study. DWU, SPH, ES, JB, KL performed biological analyses. TD, KL, ES, SPH and DWU critically drafted and revised the manuscript. All authors have read and approved the final manuscript.
